# Army Doctors in the Future

**Published:** 1900-08-04

**Authors:** 


### The Hospital

A Journal of

The Medical Sciences and Hospital Administration.

Vol. XXVIII.—No. 723.] Edited by Sir Henry Burdett, K.C.B., and Solomon C. Smith, M.D., M.R.C.P. [August 4, 1900.

### Army Doctors in the Future.

Whatever may be the issue of the inquiries to be made by the Royal Commission with regard to the condition of the army hospitals in the Orange River Colony and elsewhere in South Africa, there can be no doubt on the mind of any reasonable person that very radical changes in the organisation of the Medical Department, both at home and in the field, should be the outcome of much of the experience which has lately been gained, and which seems likely to be extended in the near future. We must not forget that a similar belief was widely entertained after the Crimean War, and that it ultimately failed of realisation; the very considerable facilities for obstruction possessed by the so-called "combatant" departments of the service, and in still greater degree by the civilian element at the War Office, having been sufficiently powerful to prolong a dribble of aimless and ineffective talk until the popular enthusiasm at first awakened had either died away or had been diverted into other channels. Our knowledge of the working of English institutions will not permit us to be exceedingly surprised if the events of the middle of the century should be repeated at its close, and if the next war in which we are engaged should find us hampered by the very same conditions which our reformers are at present eager to change. Nevertheless, we may hope for better things, and it may not be altogether fruitless labour to discuss the possible arrangements by which such hope might be fulfilled. The objects to be attained, as far as the medical department of the Army is concerned, are, in the first place, to render it completely adequate to all military demands, including those of Indian and Colonial duty, and of "little wars" in time of peace, and at the same time to provide for its spontaneous expansion to a degree sufficient to meet the requirements of even a prolonged and serious war.

It has been pointed out with sufficient frequency, and cannot indeed be reasonably denied, that the argument in favour of conferring substantive military rank upon the officers of the medical department of the army depends upon their being constantly called upon to direct the movements of considerable bodies of men by whom no other form of rank is recognised, and who cannot be effectively controlled by anything short of military discipline. The chief medical officer of an army corps of fifty thousand men will have not less than twelve hundred allotted to the performance of the work which it is his business to direct, this number being made up of subordinate medical officers, orderlies, stretcher bearers, drivers, and, in short, of all the *personnel* required for the conduct and management of base and field hospitals, and for the maintenance of communications between them. The substantive rank has now been conceded; and many of the South African war correspondents or civilian surgeons are maintaining that it can only be rendered completely effective by giving the department complete control of its own transport. This does not sound to us extremely practical; because it is certain that the wants in this direction of the medical or any other department must be subordinated in time of war to any others which, in the opinion of the Commander-in-Chief, might be of still greater importance to the conduct of his operations. In the case of an army fed by a long line of railway it is manifest that all departmental authority, as far as transport was concerned, however complete in theory, would in practice be liable to be overriden from head-quarters.

A point of supreme importance is, we think, that proper means should be adopted for rescuing army medical officers from the grooves into which the limitations of their experience must at times be apt to confine them; and there could be no better way of doing this than that which has more than once been suggested on their behalf, namely, that their numbers should be so adjusted to ordinary military requirements as to give to each one a leave on service, so to speak, at stated intervals, of sufficient length to enable him to visit hospitals, and to make himself practically acquainted with the chief improvements in medicine and surgery which the few preceding years had brought into prominence. If this could be done it would secure that the army surgeon should always be fairly abreast of the applied science of his day, and the department would be fully prepared to deal with the requirements of ordinary times. Such a provision, supplemented by one under which civil practitioners of proved ability were encouraged to enter, and to remain for ten or more years in, a sort of Medical

Reserve, receiving some small retaining pay and a title, and liable to be called upon to join the ranks in time of war or great national emergency, might be fairly expected to meet every contingency at all likely to arise, and would probably prove a better and more effective plan than that of suddenly calling eminent consultants from their work in hospital and private practice, and hastily despatching them to stop gaps which should not have been suffered to exist. With such a reserve, the members of which might be called upon to familiarise themselves, as far as possible, with military medical requirements, such needs as those which we have lately felt would be provided for by an existing and adequate organisation.

